# Heterotopic Pancreatic Tissue in Gallbladder: A Report of an Unusual Entity at an Extremely Rare Location 

**DOI:** 10.30699/ijp.2025.2019770.3244

**Published:** 2025-03-10

**Authors:** Mukta Pujani, Aparna Khandelwal, Kanika Singh, Meenu Pujani, Cherry Bansal

**Affiliations:** 1 *Department of Pathology, ESIC Medical College & Hospital, NIT-3, Faridabad, India*; 2 *SRL Diagnostic Lab, Chandigarh, India*; 3 *Department of Pathology* *, UCMS & GTB Hospital, New Delhi, India*; 4 *Metro Heart Institute & Superspecialty Hospital, Faridabad, India*; 5 *Dr S.S. Tantia Medical College, Hospital and Research Center, Sriganganagar, India*

**Keywords:** Heterotopic pancreas, ectopic pancreas, gallbladder

## Abstract

**Background & Objective:**

Heterotopic pancreas (HP) or ectopic pancreas is the occurrence of pancreatic tissue in an atypical location with absence of any neurovascular or anatomic connection with the normal pancreas. In an autopsy series, the incidence of this embryologic anomaly is 0.55% to 13.7% of patients. Gallbladder is an extremely rare site for ectopic pancreatic tissue with approximately 40 documented cases.

**Case Presentation:**

We hereby report a case of incidental discovery of ectopic pancreatic tissue in the excised gallbladder from a 27-year-old female who presented with nausea, vomiting, and abdominal pain intermittently. The gallbladder lumen was filled with biliary sludge containing a single gallstone. Histopathology revealed chronic cholecystitis along with a tiny focus of ectopic pancreatic tissue comprising only pancreatic acini.

**Conclusion:**

This case highlights that histopathology should be mandatory for all excised gallbladder specimens and that this entity should be considered among the differentials for nodular/polypoidal gallbladder lesions. Although the cases where the ectopic pancreas is discovered incidentally do not have much clinical significance, this may prevent the patient from undergoing more aggressive treatment reserved for conditions like pancreatitis or malignancy in cases where the ectopic pancreas mimics a malignancy.

## Introduction

Heterotopic pancreas (HP) is the presence of pancreatic tissue in an atypical location anatomically distant from the normal pancreas. The usual sites of occurrence include the stomach, duodenum, jejunum, Meckel diverticulum, and mesentery (1); however, the gallbladder is a very rare site with only approximately 40 cases described in the literature, comprising approximately 1% of all heterotopic pancreas.

## Case description

A 27-year-old female presented to the outpatient clinics with complaints of nausea, vomiting, and abdominal pain intermittently for the last 3 months, aggravated following meals. The right hypochondrium was tender with a positive Murphy sign. Abdominal ultrasound revealed a gallbladder lumen filled with biliary sludge containing a single gallstone and minimal wall edema. Her hematologic, liver, and kidney function tests were within normal limits. Laparoscopic cholecystectomy was carried out with a provisional diagnosis of acute cholecystitis with cholelithiasis. 

Grossly, the gallbladder measured 8.5 cm × 6 cm, and the wall thickness ranged from 0.2 to 0.4 cm. No nodule, polyp, or growth was identified. Thick biliary sludge and a single stone approximately 1.2 cm in diameter were seen in the lumen. 

Microscopic examination revealed chronic cholecystitis along with a tiny focus of ectopic pancreatic tissue in the subepithelial area consisting of pancreatic acini only, without any ducts or islet cells (Figures 1). 

The patient’s follow-up for a period of 2 years post-surgery was unremarkable. 

**Fig.**
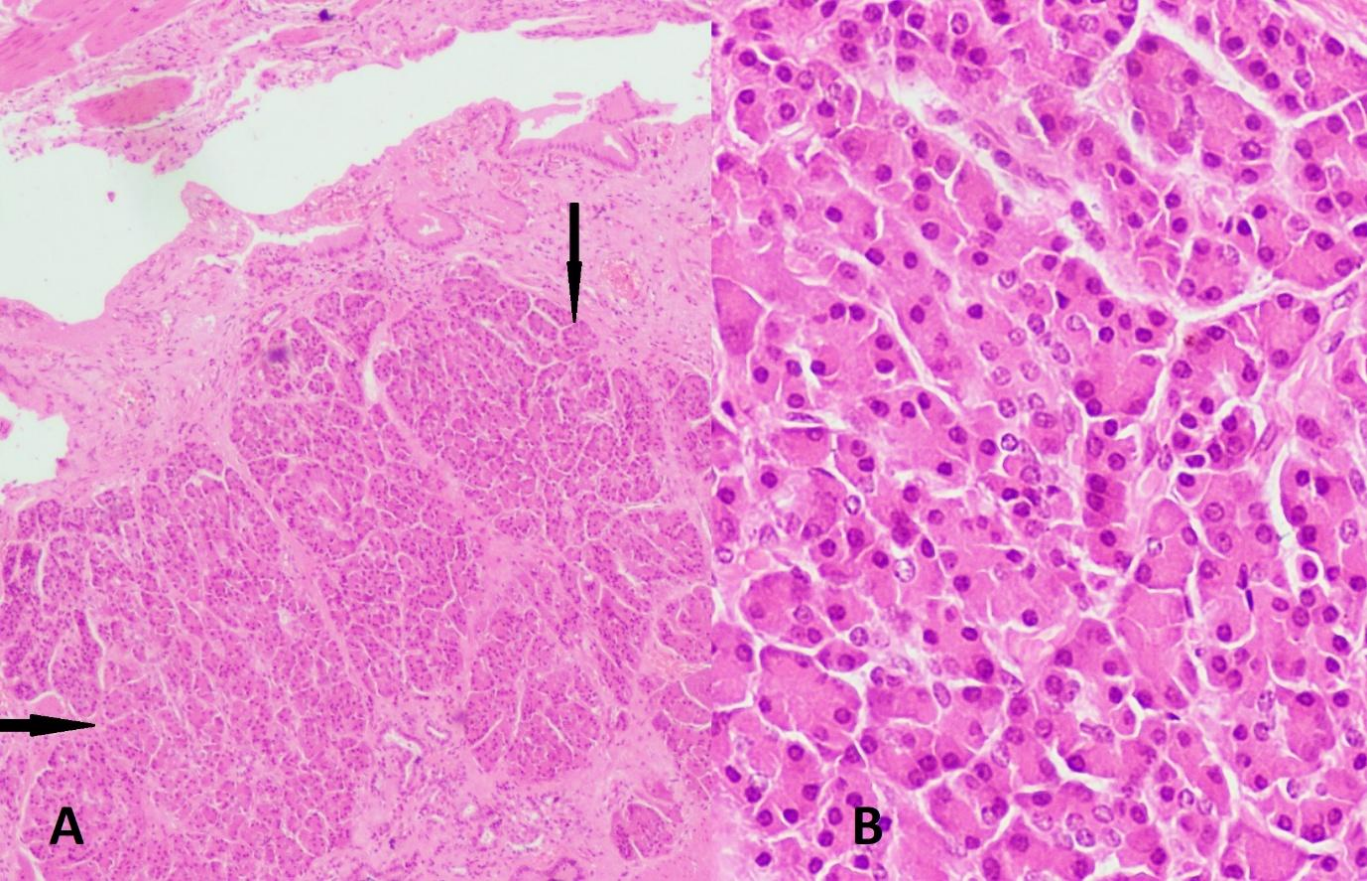
A) Section showing gallbladder lining with the ectopic pancreatic tissue (black arrows) in the subepithelial location (Hematoxylin & Eosin, ×100); B) Heterotopic pancreas tissue comprising only acini (Hematoxylin & Eosin, ×400)

## Discussion

Heterotopic pancreas (HP), ectopic pancreas, pancreatic rest, or pancreatic choristoma is the occurrence of pancreatic tissue in an abnormal location without any neurovascular or anatomic connection with the main pancreas at the normal position. The incidence of this embryologic anomaly is 0.55% to 13.7% of patients in an autopsy series (3). The gallbladder is a scarce site for ectopic pancreatic tissue. It was reported for the first time in the gallbladder in the early 1900s by Otschkin. An extensive review of the literature shows that approximately 40 cases have been documented in the gallbladder, accounting for 1% of all heterotopic pancreas (3). 

Although the embryologic basis of the occurrence of ectopic pancreas is not clearly understood, several proposed theories to explain the phenomenon include misplacement or metaplasia or stem cell theories. Among these, the misplacement theory is the most well-accepted and states that during development, pancreatic cell populations are “dropped” into the gastrointestinal system (GIT), anatomically away from the main pancreas, thereby explaining the most common locations for the heterotopic pancreas (3). 

Incidental detection of heterotopic pancreas is the typical clinical presentation during an unrelated surgery, during radiologic examination, or at autopsy. Most patients with heterotopic pancreatic tissue have nonspecific symptoms pertaining to coexisting cholecystitis and/or cholelithiasis, including anorexia, upper abdominal pain, nausea, vomiting after meals, and weight loss, etc. (2,4,8,10). However, ectopic pancreatic tissue may be associated with complications like acute pancreatitis or the development of dysplasia or cancer (5). 

Heterotopic pancreatic tissue in the gallbladder is usually found in the neck and body, perhaps due to the close proximity of these areas to the lateral pancreas during development. The lesions range from 4 to 20 mm in size and may present as nodules or polyps. Most heterotopic pancreatic lesions of the gallbladder are submucosal, followed by intramuscular and subserosal locations. In the present case, the lesion was also observed in the neck region, submucosally (3). 

Diagnosis of hepatobiliary heterotopic pancreas is rendered on histopathology. Pancreatic acini and ducts are observed most commonly, while only one third of cases have documented presence of islet cells. A classification of ectopic pancreatic tissue on histopathology was proposed by von Heinrich (6) and later modified by Gaspar Fuentes et al (7) in 1973. It further categorizes heterotopic pancreatic tissue as follows: Type 1: the presence of acini, ducts, and islet-like pancreatic gland; Type 2: canalicular variant with pancreatic ducts; Type 3: exocrine pancreas with acinar tissue; and Type 4: endocrine pancreas with cellular islets. Our case belonged to type 3. 

## Conclusion

Heterotopic pancreatic tissue in the gallbladder is an extremely unusual embryologic anomaly detected incidentally in most cases, thereby stressing the fact that histopathology should be mandatory for all excised gallbladder specimens. Moreover, this entity ought to be considered in the differential diagnosis of nodular/polypoidal lesions of the gallbladder, especially acalculous cases or those coexisting with raised amylase of uncertain origin. Although the cases where ectopic pancreas is discovered incidentally do not have much clinical significance, this may prevent the patient from undergoing more aggressive treatment reserved for conditions like pancreatitis or malignancy in cases where the ectopic pancreas mimics a malignancy. 

## Data Availability

Data are available upon reasonable request from the corresponding author.
